# Bis(4-aminobenzenesulfonato)tri­aqua­bis(1,10-phenanthroline)neodymium(III) nitrate tetrahydrate

**DOI:** 10.1107/S1600536810023445

**Published:** 2010-06-23

**Authors:** Peng Ma, Miao-Ling Huang, Kai-Han Chen

**Affiliations:** a Department of Chemistry and Science of Life, Quanzhou Normal University, Fujian 362000, People’s Republic of China

## Abstract

The title complex, [Nd(C_6_H_6_NO_3_S)_2_(C_12_H_8_N_2_)_2_(H_2_O)_3_]NO_3_·4H_2_O, comprises a mononuclear cation, an NO_3_^−^ anion and two uncoordinated water mol­ecules; the Nd^III^ cation, one coordinated water mol­ecule, and the NO_3_^−^ anion each lie on a twofold axis of symmetry. The Nd^III^ ion exhibits an NdN_4_O_5_ coordination environment comprising two O atoms of two monodentate 4-amino­benzene­sulfonato ligands, four N atoms of the bidentate 1,10-phenanthroline ligands, and three water-O atoms. The coordination geometry is based on a tricapped triangular-prismatic arrangement. The components are consolidated into a three-dimensional network *via* O—H⋯O, O—H⋯N and N—H⋯O hydrogen-bonding inter­actions

## Related literature

For background to the applications of rare earth complexes, see: Li *et al.* (2007 ▶); Tang *et al.* (2006 ▶); Xie *et al.* (2009 ▶).
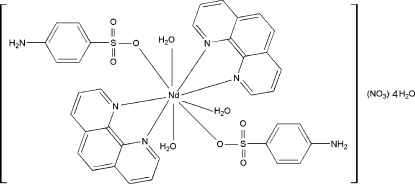


## Experimental

### 

#### Crystal data

[Nd(C_6_H_6_NO_3_S)_2_(C_12_H_8_N_2_)_2_(H_2_O)_3_]NO_3_·4H_2_O
*M* *_r_* = 1037.13
Orthorhombic, 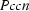

*a* = 17.4990 (18) Å
*b* = 14.2788 (15) Å
*c* = 16.7045 (17) Å
*V* = 4173.9 (7) Å^3^
*Z* = 4
Mo *K*α radiation
μ = 1.42 mm^−1^
*T* = 293 K
0.32 × 0.22 × 0.16 mm

#### Data collection

Bruker SMART CCD area-detector diffractometer
Absorption correction: multi-scan (*SADABS*; Sheldrick, 2003 ▶) *T*_min_ = 0.656, *T*_max_ = 0.789
34751 measured reflections
3883 independent reflections
3287 reflections with *I* > 2σ(*I*)
*R*_int_ = 0.027

#### Refinement

*R*[*F* ^2^ > 2σ(*F* ^2^)] = 0.026
*wR*(*F* ^2^) = 0.066
*S* = 1.21
3883 reflections
282 parameters
H-atom parameters constrained
Δρ_max_ = 0.31 e Å^−3^
Δρ_min_ = −0.76 e Å^−3^

### 

Data collection: *SMART* (Bruker, 2001 ▶); cell refinement: *SAINT* (Bruker, 2003 ▶); data reduction: *SAINT*; program(s) used to solve structure: *SHELXTL* (Sheldrick, 2008 ▶); program(s) used to refine structure: *SHELXTL*; molecular graphics: *SHELXTL*; software used to prepare material for publication: *SHELXTL*.

## Supplementary Material

Crystal structure: contains datablocks global, I. DOI: 10.1107/S1600536810023445/tk2665sup1.cif

Structure factors: contains datablocks I. DOI: 10.1107/S1600536810023445/tk2665Isup2.hkl

Additional supplementary materials:  crystallographic information; 3D view; checkCIF report

## Figures and Tables

**Table 1:** Selected bond lengths (Å)

Nd1—O1	2.4007 (18)
Nd1—O5	2.462 (2)
Nd1—O4	2.528 (3)
Nd1—N3	2.703 (2)
Nd1—N2	2.763 (2)

**Table 2:** Hydrogen-bond geometry (Å, °)

*D*—H⋯*A*	*D*—H	H⋯*A*	*D*⋯*A*	*D*—H⋯*A*
O9—H6*W*⋯O3	0.85	1.97	2.810 (3)	171
O8—H5*W*⋯N4	0.86	2.61	3.391 (3)	152
O8—H5*W*⋯O7	0.86	2.53	3.109 (4)	126
O8—H5*W*⋯O6	0.86	1.99	2.842 (3)	170
O8—H4*W*⋯O3	0.83	2.03	2.861 (3)	174
O9—H7*W*⋯N1^i^	0.86	2.24	2.982 (4)	145
O5—H3*W*⋯O8^ii^	0.85	1.96	2.793 (3)	168
O5—H2*W*⋯O9^iii^	0.85	1.83	2.670 (3)	171
O4—H1*W*⋯O2^iii^	0.83	1.95	2.773 (3)	171
N1—H1*A*⋯O8^iv^	0.89	2.29	3.127 (4)	158

Symmetry codes: (i) 
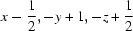
; (ii) 
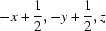
; (iii) 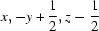; (iv) 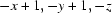.

**Fig. 1.**
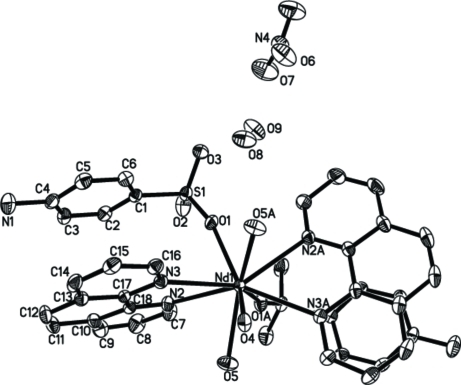
Molecular structures of the components of (I) showing the atom-labelling scheme and displacement ellipsoids at the 50% probability level. Hydrogen atoms have been omitted for reasons of clarity. Symmetry operation a: 1-x, 1-y, -z.

## supplementary crystallographic information

### Comment

Rare earth complexes have been extensively studied owing to their unique structures and potential applications as biomedical, catalytic, and magnetic agents, as well as nonlinear optical materials (Tang *et al.*, 2006; Li *et al.*, 2007). Therefore, the rational design and synthesis of rare earth supramolecular complexes are highlighted in supramolecular and biochemical research.

The molecules comprising the asymmetric unit of the title compound, (I), are presented in Fig 1. The structure comprises a mononuclear [Nd(C_6_H_6_NO_3_S)_2_(C_12_H_8_N_2_)_2_(H_2_O)_3_]^+^ cation, one NO_3_^-^ anion and two lattice water molecules. The Nd(III) cation, one coordinated water molecule (O4) and the NO_3_^-^ anion are each located on a crystallographic 2-fold axis. The Nd(III) ion is within a distorted tricapped triangular prismatic coordination polyhedron completed by two O atoms from two 4-aminobenzenesulfonato ligands, three O atoms from three coordinated water, and four N atom from two 1,10-phenanthroline ligands; see Table 1 for bond distances. The average bond lengths of Nd—O and Nd—N are shorter than the averages of the comparable bond lengths in [Ln(C_6_H_6_NO_3_S)_2_(C_12_H_8_N_2_)_2_(H_2_O)_3_]NO_3_.(H_2_O)_2_ for Ln = La^III^ and Ce^III^ (Xie *et al.*, 2009), consistent with the lanthanide contraction.

The components of the structure are consolidated into a 3-D network via hydrogen bonding interactions, Table 2.

### Experimental

An aqueous solution (5 ml) of Nd(NO_3_)_3_.6H_2_O (1.0 mmol) was added slowly to a solution of *p*-aminobenzenesulfonilic acid (1.0 mmol) in H_2_O (5 ml). After refluxing for 2 h, a solution of 1,10-phenanthroline (1.0 mmol) in ethanol (95%, 5 ml) was added slowly to the solution. Refluxing was continued for 2 h followed by filteration of the hot mixture. The purple single crystals suitable for X-ray analysis were obtained after three weeks by slow evaporation of the above filtrate held at room temperature. Yield 52%. IR (KBr): 3425(vs), 1666(s), 1624(s), 1600(s), 1572(m), 1515(s), 1506(s), 1419(vs), 1384(vs), 1342(w), 1316(m), 1304(m), 1231(s), 1122(vs), 1039(vs), 1012(s), 848(s), 825(m), 776(m), 729(s), 700(s), 632(m), 574(s) cm^-1^.

### Refinement

H atoms bonded to N and C were placed geometrically and treated as riding, (N–H = 0.89 Å and C—H = 0.93 Å), with U_iso_(H) = 1.5U_eq_(N) or 1.2U_eq_(C). The water-bound H atoms were found from Fourier difference maps, fixed at these positions (0.83-0.86 Å), and were refined as riding with U_iso_(H) = 1.5U_eq_(O).

### Crystal data

**Table:**

[Nd(C_6_H_6_NO_3_S)_2_(C_12_H_8_N_2_)_2_(H_2_O)_3_]NO_3_·4H_2_O	*F*(000) = 2108
*M**_r_* = 1037.13	*D*_x_ = 1.650 Mg m^−^^3^
Orthorhombic, *P**c**c**n*	Mo *K*α radiation, λ = 0.71073 Å
Hall symbol: -P 2ab 2ac	Cell parameters from 2148 reflections
*a* = 17.4990 (18) Å	θ = 2.5–23.3°
*b* = 14.2788 (15) Å	µ = 1.42 mm^−^^1^
*c* = 16.7045 (17) Å	*T* = 293 K
*V* = 4173.9 (7) Å^3^	Block, pink
*Z* = 4	0.32 × 0.22 × 0.16 mm

### Data collection

**Table:**

Bruker SMART CCD area-detector diffractometer	3883 independent reflections
Radiation source: fine-focus sealed tube	3287 reflections with *I* > 2σ(*I*)
graphite	*R*_int_ = 0.027
φ and ω scans	θ_max_ = 25.5°, θ_min_ = 3.0°
Absorption correction: multi-scan (*SADABS*; Sheldrick, 2003)	*h* = −21→21
*T*_min_ = 0.656, *T*_max_ = 0.789	*k* = −17→17
34751 measured reflections	*l* = −20→20

### Refinement

**Table:**

Refinement on *F*^2^	Primary atom site location: structure-invariant direct methods
Least-squares matrix: full	Secondary atom site location: difference Fourier map
*R*[*F*^2^ > 2σ(*F*^2^)] = 0.026	Hydrogen site location: inferred from neighbouring sites
*wR*(*F*^2^) = 0.066	H-atom parameters constrained
*S* = 1.21	*w* = 1/[σ^2^(*F*_o_^2^) + (0.0264*P*)^2^ + 3.2531*P*] where *P* = (*F*_o_^2^ + 2*F*_c_^2^)/3
3883 reflections	(Δ/σ)_max_ = 0.001
282 parameters	Δρ_max_ = 0.31 e Å^−^^3^
0 restraints	Δρ_min_ = −0.76 e Å^−^^3^

### Special details

**Table:**

Geometry. All esds (except the esd in the dihedral angle between two l.s. planes) are estimated using the full covariance matrix. The cell esds are taken into account individually in the estimation of esds in distances, angles and torsion angles; correlations between esds in cell parameters are only used when they are defined by crystal symmetry. An approximate (isotropic) treatment of cell esds is used for estimating esds involving l.s. planes.
Refinement. Refinement of *F*^2^ against ALL reflections. The weighted *R*-factor wR and goodness of fit *S* are based on *F*^2^, conventional *R*-factors *R* are based on *F*, with *F* set to zero for negative *F*^2^. The threshold expression of *F*^2^ > σ(*F*^2^) is used only for calculating *R*-factors(gt) etc. and is not relevant to the choice of reflections for refinement. *R*-factors based on *F*^2^ are statistically about twice as large as those based on *F*, and *R*- factors based on ALL data will be even larger.

### Fractional atomic coordinates and isotropic or equivalent isotropic displacement parameters (Å^2^)

**Table:**

	*x*	*y*	*z*	*U*_iso_*/*U*_eq_	
Nd1	0.2500	0.2500	0.038262 (10)	0.02429 (7)	
S1	0.36306 (4)	0.36807 (5)	0.20699 (4)	0.03383 (16)	
O1	0.31177 (10)	0.33811 (14)	0.14217 (11)	0.0411 (5)	
O2	0.35809 (13)	0.3088 (2)	0.27640 (12)	0.0611 (7)	
O3	0.35040 (13)	0.46714 (16)	0.22412 (13)	0.0529 (6)	
O4	0.2500	0.2500	−0.11307 (15)	0.0374 (6)	
H1W	0.2862	0.2332	−0.1422	0.056*	
O5	0.24704 (12)	0.08714 (15)	−0.01000 (13)	0.0483 (5)	
H2W	0.2473	0.0658	−0.0576	0.073*	
H3W	0.2417	0.0418	0.0226	0.073*	
O6	0.2500	0.7500	0.1311 (3)	0.0821 (12)	
H4W	0.3105	0.5399	0.1283	0.123*	
H5W	0.2734	0.6180	0.1017	0.123*	
O7	0.2625 (2)	0.6779 (2)	0.2436 (2)	0.0967 (10)	
H6W	0.2805	0.4911	0.3142	0.145*	
H7W	0.2402	0.5571	0.3568	0.145*	
O8	0.29153 (17)	0.56444 (16)	0.08766 (14)	0.0662 (7)	
O9	0.24465 (15)	0.49835 (19)	0.34785 (14)	0.0708 (8)	
N1	0.67660 (15)	0.3332 (2)	0.0745 (2)	0.0696 (9)	
H1A	0.6763	0.3506	0.0233	0.104*	
H1B	0.6911	0.2736	0.0780	0.104*	
N2	0.36395 (12)	0.14185 (16)	0.10577 (13)	0.0346 (5)	
N3	0.39407 (13)	0.26033 (15)	−0.01948 (13)	0.0311 (5)	
N4	0.2500	0.7500	0.2066 (3)	0.0571 (11)	
C1	0.45674 (14)	0.35885 (18)	0.16953 (14)	0.0292 (6)	
C2	0.50570 (16)	0.2901 (2)	0.19801 (16)	0.0371 (6)	
H2	0.4895	0.2495	0.2381	0.045*	
C3	0.57842 (17)	0.2820 (2)	0.1670 (2)	0.0450 (7)	
H3	0.6113	0.2365	0.1871	0.054*	
C4	0.60297 (16)	0.3411 (2)	0.10621 (18)	0.0426 (7)	
C5	0.55417 (17)	0.4112 (2)	0.07957 (17)	0.0422 (7)	
H5	0.5707	0.4528	0.0404	0.051*	
C6	0.48166 (16)	0.4196 (2)	0.11059 (16)	0.0370 (6)	
H6	0.4493	0.4663	0.0918	0.044*	
C7	0.35188 (18)	0.0874 (2)	0.16914 (19)	0.0480 (8)	
H7	0.3047	0.0908	0.1946	0.058*	
C8	0.4070 (2)	0.0249 (3)	0.1995 (2)	0.0576 (9)	
H8	0.3959	−0.0118	0.2441	0.069*	
C9	0.4759 (2)	0.0187 (2)	0.1636 (2)	0.0548 (9)	
H9	0.5125	−0.0230	0.1827	0.066*	
C10	0.49175 (16)	0.0758 (2)	0.09720 (18)	0.0423 (7)	
C11	0.56503 (18)	0.0738 (3)	0.0574 (2)	0.0541 (9)	
H11	0.6023	0.0318	0.0743	0.065*	
C12	0.57972 (17)	0.1318 (3)	−0.0037 (2)	0.0528 (9)	
H12	0.6271	0.1293	−0.0288	0.063*	
C13	0.52349 (16)	0.1980 (2)	−0.03103 (17)	0.0397 (7)	
C14	0.53933 (17)	0.2646 (2)	−0.09115 (18)	0.0467 (8)	
H14	0.5869	0.2657	−0.1159	0.056*	
C15	0.48461 (17)	0.3270 (2)	−0.11248 (17)	0.0427 (7)	
H15	0.4948	0.3727	−0.1506	0.051*	
C16	0.41244 (16)	0.3217 (2)	−0.07639 (16)	0.0361 (6)	
H16	0.3751	0.3636	−0.0932	0.043*	
C17	0.44996 (14)	0.19932 (19)	0.00458 (16)	0.0305 (6)	
C18	0.43377 (15)	0.13720 (18)	0.07021 (16)	0.0319 (6)	

### Atomic displacement parameters (Å^2^)

**Table:**

	*U*^11^	*U*^22^	*U*^33^	*U*^12^	*U*^13^	*U*^23^
Nd1	0.02232 (11)	0.02820 (11)	0.02233 (11)	0.00157 (8)	0.000	0.000
S1	0.0281 (3)	0.0459 (4)	0.0275 (3)	−0.0015 (3)	−0.0001 (3)	−0.0048 (3)
O1	0.0293 (10)	0.0500 (12)	0.0442 (11)	−0.0011 (9)	−0.0082 (8)	−0.0131 (10)
O2	0.0438 (13)	0.099 (2)	0.0406 (12)	0.0019 (13)	0.0119 (10)	0.0245 (12)
O3	0.0455 (13)	0.0524 (13)	0.0607 (14)	0.0027 (10)	0.0018 (10)	−0.0276 (11)
O4	0.0294 (13)	0.0596 (18)	0.0231 (13)	0.0086 (12)	0.000	0.000
O5	0.0782 (16)	0.0345 (11)	0.0323 (10)	0.0046 (10)	−0.0075 (10)	−0.0015 (9)
O6	0.112 (4)	0.070 (3)	0.064 (3)	0.030 (2)	0.000	0.000
O7	0.136 (3)	0.0635 (19)	0.091 (2)	0.0118 (18)	0.003 (2)	0.0209 (19)
O8	0.095 (2)	0.0502 (14)	0.0531 (14)	0.0019 (14)	0.0048 (14)	−0.0095 (12)
O9	0.099 (2)	0.0661 (17)	0.0477 (14)	0.0286 (14)	0.0217 (13)	0.0124 (13)
N1	0.0397 (16)	0.081 (2)	0.088 (2)	−0.0044 (15)	0.0208 (16)	−0.0120 (19)
N2	0.0288 (12)	0.0390 (13)	0.0359 (12)	0.0026 (10)	−0.0036 (10)	0.0035 (10)
N3	0.0296 (12)	0.0345 (13)	0.0291 (11)	−0.0014 (9)	−0.0006 (9)	−0.0023 (10)
N4	0.055 (3)	0.052 (3)	0.064 (3)	0.0073 (19)	0.000	0.000
C1	0.0276 (13)	0.0366 (15)	0.0233 (12)	−0.0038 (11)	−0.0015 (10)	−0.0030 (11)
C2	0.0365 (16)	0.0398 (15)	0.0352 (15)	−0.0041 (13)	−0.0041 (12)	0.0061 (13)
C3	0.0324 (16)	0.0465 (17)	0.0559 (19)	0.0029 (13)	−0.0060 (14)	0.0006 (15)
C4	0.0315 (15)	0.0512 (18)	0.0449 (17)	−0.0100 (13)	0.0041 (13)	−0.0140 (15)
C5	0.0458 (17)	0.0458 (17)	0.0349 (15)	−0.0116 (14)	0.0068 (13)	0.0031 (14)
C6	0.0400 (16)	0.0374 (15)	0.0337 (14)	−0.0013 (12)	−0.0018 (12)	0.0031 (12)
C7	0.0408 (17)	0.056 (2)	0.0474 (18)	0.0022 (15)	−0.0044 (14)	0.0198 (16)
C8	0.057 (2)	0.060 (2)	0.056 (2)	0.0021 (17)	−0.0161 (17)	0.0238 (17)
C9	0.052 (2)	0.0476 (19)	0.065 (2)	0.0147 (15)	−0.0201 (17)	0.0099 (17)
C10	0.0363 (16)	0.0383 (16)	0.0523 (18)	0.0098 (13)	−0.0122 (13)	−0.0072 (14)
C11	0.0380 (18)	0.056 (2)	0.068 (2)	0.0207 (15)	−0.0120 (16)	−0.0133 (18)
C12	0.0272 (15)	0.068 (2)	0.064 (2)	0.0101 (15)	0.0037 (15)	−0.0190 (19)
C13	0.0302 (15)	0.0477 (18)	0.0412 (16)	−0.0022 (13)	0.0007 (12)	−0.0165 (14)
C14	0.0310 (15)	0.069 (2)	0.0399 (16)	−0.0109 (15)	0.0081 (13)	−0.0170 (16)
C15	0.0450 (17)	0.0521 (18)	0.0312 (14)	−0.0155 (14)	0.0048 (13)	−0.0052 (14)
C16	0.0361 (15)	0.0407 (16)	0.0315 (14)	−0.0054 (12)	−0.0002 (12)	−0.0016 (13)
C17	0.0242 (13)	0.0335 (15)	0.0337 (14)	0.0001 (11)	−0.0038 (11)	−0.0110 (12)
C18	0.0283 (14)	0.0320 (14)	0.0354 (14)	0.0015 (11)	−0.0072 (11)	−0.0072 (12)

### Geometric parameters (Å, °)

**Table:**

Nd1—O1	2.4007 (18)	C1—C2	1.387 (4)
Nd1—O1^i^	2.4007 (18)	C1—C6	1.383 (4)
Nd1—O5^i^	2.462 (2)	C2—C3	1.379 (4)
Nd1—O5	2.462 (2)	C2—H2	0.9300
Nd1—O4	2.528 (3)	C3—C4	1.389 (4)
Nd1—N3^i^	2.703 (2)	C3—H3	0.9300
Nd1—N3	2.703 (2)	C4—C5	1.389 (4)
Nd1—N2	2.763 (2)	C5—C6	1.376 (4)
Nd1—N2^i^	2.763 (2)	C5—H5	0.9300
S1—O2	1.438 (2)	C6—H6	0.9300
S1—O3	1.460 (2)	C7—C8	1.408 (4)
S1—O1	1.4701 (18)	C7—H7	0.9300
S1—C1	1.760 (3)	C8—C9	1.350 (5)
O4—H1W	0.8343	C8—H8	0.9300
O5—H2W	0.8517	C9—C10	1.404 (4)
O5—H3W	0.8502	C9—H9	0.9300
O6—N4	1.262 (6)	C10—C18	1.415 (4)
O7—N4	1.220 (4)	C10—C11	1.445 (4)
O8—H4W	0.8327	C11—C12	1.339 (5)
O8—H5W	0.8602	C11—H11	0.9300
O9—H6W	0.8478	C12—C13	1.439 (4)
O9—H7W	0.8562	C12—H12	0.9300
N1—C4	1.398 (4)	C13—C14	1.411 (4)
N1—H1A	0.8900	C13—C17	1.418 (4)
N1—H1B	0.8900	C14—C15	1.355 (4)
N2—C7	1.330 (4)	C14—H14	0.9300
N2—C18	1.360 (3)	C15—C16	1.401 (4)
N3—C16	1.332 (3)	C15—H15	0.9300
N3—C17	1.370 (3)	C16—H16	0.9300
N4—O7^ii^	1.220 (4)	C17—C18	1.438 (4)
			
O1—Nd1—O1^i^	87.40 (10)	O7^ii^—N4—O7	119.2 (5)
O1—Nd1—O5^i^	74.47 (7)	O7^ii^—N4—O6	120.4 (3)
O1^i^—Nd1—O5^i^	137.84 (7)	O7—N4—O6	120.4 (3)
O1—Nd1—O5	137.84 (7)	C2—C1—C6	119.6 (2)
O1^i^—Nd1—O5	74.47 (7)	C2—C1—S1	120.4 (2)
O5^i^—Nd1—O5	141.77 (10)	C6—C1—S1	120.0 (2)
O1—Nd1—O4	136.30 (5)	C1—C2—C3	120.1 (3)
O1^i^—Nd1—O4	136.30 (5)	C1—C2—H2	120.0
O5^i^—Nd1—O4	70.89 (5)	C3—C2—H2	120.0
O5—Nd1—O4	70.89 (5)	C2—C3—C4	120.6 (3)
O1—Nd1—N3^i^	134.94 (6)	C2—C3—H3	119.7
O1^i^—Nd1—N3^i^	79.02 (6)	C4—C3—H3	119.7
O5^i^—Nd1—N3^i^	87.38 (7)	C5—C4—C3	118.8 (3)
O5—Nd1—N3^i^	79.16 (7)	C5—C4—N1	120.2 (3)
O4—Nd1—N3^i^	69.10 (5)	C3—C4—N1	120.9 (3)
O1—Nd1—N3	79.02 (6)	C6—C5—C4	120.6 (3)
O1^i^—Nd1—N3	134.94 (6)	C6—C5—H5	119.7
O5^i^—Nd1—N3	79.16 (7)	C4—C5—H5	119.7
O5—Nd1—N3	87.38 (7)	C5—C6—C1	120.3 (3)
O4—Nd1—N3	69.10 (5)	C5—C6—H6	119.9
N3^i^—Nd1—N3	138.20 (9)	C1—C6—H6	119.9
O1—Nd1—N2	70.91 (7)	N2—C7—C8	123.3 (3)
O1^i^—Nd1—N2	74.75 (6)	N2—C7—H7	118.4
O5^i^—Nd1—N2	130.28 (7)	C8—C7—H7	118.4
O5—Nd1—N2	67.72 (7)	C9—C8—C7	119.5 (3)
O4—Nd1—N2	114.09 (5)	C9—C8—H8	120.2
N3^i^—Nd1—N2	142.02 (6)	C7—C8—H8	120.2
N3—Nd1—N2	60.20 (7)	C8—C9—C10	119.3 (3)
O1—Nd1—N2^i^	74.75 (6)	C8—C9—H9	120.3
O1^i^—Nd1—N2^i^	70.91 (7)	C10—C9—H9	120.3
O5^i^—Nd1—N2^i^	67.72 (7)	C9—C10—C18	118.0 (3)
O5—Nd1—N2^i^	130.28 (7)	C9—C10—C11	121.9 (3)
O4—Nd1—N2^i^	114.09 (5)	C18—C10—C11	120.1 (3)
N3^i^—Nd1—N2^i^	60.20 (7)	C12—C11—C10	120.6 (3)
N3—Nd1—N2^i^	142.02 (6)	C12—C11—H11	119.7
N2—Nd1—N2^i^	131.82 (9)	C10—C11—H11	119.7
O2—S1—O3	113.77 (15)	C11—C12—C13	121.1 (3)
O2—S1—O1	112.68 (14)	C11—C12—H12	119.4
O3—S1—O1	109.49 (12)	C13—C12—H12	119.4
O2—S1—C1	107.40 (13)	C14—C13—C17	117.9 (3)
O3—S1—C1	106.48 (13)	C14—C13—C12	122.3 (3)
O1—S1—C1	106.57 (11)	C17—C13—C12	119.8 (3)
S1—O1—Nd1	163.72 (13)	C15—C14—C13	119.4 (3)
Nd1—O4—H1W	125.7	C15—C14—H14	120.3
Nd1—O5—H2W	130.1	C13—C14—H14	120.3
Nd1—O5—H3W	120.8	C14—C15—C16	119.2 (3)
H2W—O5—H3W	109.0	C14—C15—H15	120.4
H4W—O8—H5W	107.4	C16—C15—H15	120.4
H6W—O9—H7W	107.6	N3—C16—C15	124.0 (3)
C4—N1—H1A	109.6	N3—C16—H16	118.0
C4—N1—H1B	108.3	C15—C16—H16	118.0
H1A—N1—H1B	109.5	N3—C17—C13	122.2 (3)
C7—N2—C18	117.5 (2)	N3—C17—C18	118.4 (2)
C7—N2—Nd1	122.47 (18)	C13—C17—C18	119.4 (2)
C18—N2—Nd1	119.83 (17)	N2—C18—C10	122.4 (3)
C16—N3—C17	117.1 (2)	N2—C18—C17	118.7 (2)
C16—N3—Nd1	121.03 (18)	C10—C18—C17	118.9 (2)
C17—N3—Nd1	121.76 (17)		
			
O2—S1—O1—Nd1	−61.6 (5)	O1—S1—C1—C6	69.4 (2)
O3—S1—O1—Nd1	170.7 (4)	C6—C1—C2—C3	−0.7 (4)
C1—S1—O1—Nd1	55.9 (5)	S1—C1—C2—C3	178.5 (2)
O1^i^—Nd1—O1—S1	77.3 (4)	C1—C2—C3—C4	−1.2 (4)
O5^i^—Nd1—O1—S1	−141.2 (5)	C2—C3—C4—C5	2.8 (4)
O5—Nd1—O1—S1	14.0 (5)	C2—C3—C4—N1	−179.9 (3)
O4—Nd1—O1—S1	−102.7 (4)	C3—C4—C5—C6	−2.6 (4)
N3^i^—Nd1—O1—S1	148.9 (4)	N1—C4—C5—C6	−179.9 (3)
N3—Nd1—O1—S1	−59.5 (4)	C4—C5—C6—C1	0.8 (4)
N2—Nd1—O1—S1	2.5 (4)	C2—C1—C6—C5	0.9 (4)
N2^i^—Nd1—O1—S1	148.2 (5)	S1—C1—C6—C5	−178.3 (2)
O1—Nd1—N2—C7	88.4 (2)	C18—N2—C7—C8	−1.2 (5)
O1^i^—Nd1—N2—C7	−4.2 (2)	Nd1—N2—C7—C8	173.5 (3)
O5^i^—Nd1—N2—C7	136.7 (2)	N2—C7—C8—C9	0.0 (5)
O5—Nd1—N2—C7	−83.3 (2)	C7—C8—C9—C10	0.9 (5)
O4—Nd1—N2—C7	−138.5 (2)	C8—C9—C10—C18	−0.6 (5)
N3^i^—Nd1—N2—C7	−52.1 (3)	C8—C9—C10—C11	178.6 (3)
N3—Nd1—N2—C7	176.5 (2)	C9—C10—C11—C12	−177.1 (3)
N2^i^—Nd1—N2—C7	41.5 (2)	C18—C10—C11—C12	2.1 (5)
O1—Nd1—N2—C18	−96.98 (19)	C10—C11—C12—C13	0.4 (5)
O1^i^—Nd1—N2—C18	170.5 (2)	C11—C12—C13—C14	175.2 (3)
O5^i^—Nd1—N2—C18	−48.6 (2)	C11—C12—C13—C17	−2.9 (5)
O5—Nd1—N2—C18	91.31 (19)	C17—C13—C14—C15	0.2 (4)
O4—Nd1—N2—C18	36.1 (2)	C12—C13—C14—C15	−177.9 (3)
N3^i^—Nd1—N2—C18	122.54 (18)	C13—C14—C15—C16	−2.3 (4)
N3—Nd1—N2—C18	−8.93 (17)	C17—N3—C16—C15	0.1 (4)
N2^i^—Nd1—N2—C18	−143.9 (2)	Nd1—N3—C16—C15	−175.9 (2)
O1—Nd1—N3—C16	−101.0 (2)	C14—C15—C16—N3	2.3 (4)
O1^i^—Nd1—N3—C16	−176.01 (18)	C16—N3—C17—C13	−2.5 (4)
O5^i^—Nd1—N3—C16	−24.94 (19)	Nd1—N3—C17—C13	173.55 (18)
O5—Nd1—N3—C16	119.1 (2)	C16—N3—C17—C18	175.2 (2)
O4—Nd1—N3—C16	48.57 (18)	Nd1—N3—C17—C18	−8.8 (3)
N3^i^—Nd1—N3—C16	48.57 (18)	C14—C13—C17—N3	2.3 (4)
N2—Nd1—N3—C16	−175.2 (2)	C12—C13—C17—N3	−179.5 (3)
N2^i^—Nd1—N3—C16	−54.2 (2)	C14—C13—C17—C18	−175.3 (2)
O1—Nd1—N3—C17	83.12 (19)	C12—C13—C17—C18	2.8 (4)
O1^i^—Nd1—N3—C17	8.1 (2)	C7—N2—C18—C10	1.5 (4)
O5^i^—Nd1—N3—C17	159.2 (2)	Nd1—N2—C18—C10	−173.37 (19)
O5—Nd1—N3—C17	−56.76 (19)	C7—N2—C18—C17	−176.3 (3)
O4—Nd1—N3—C17	−127.28 (19)	Nd1—N2—C18—C17	8.8 (3)
N3^i^—Nd1—N3—C17	−127.28 (19)	C9—C10—C18—N2	−0.6 (4)
N2—Nd1—N3—C17	8.96 (17)	C11—C10—C18—N2	−179.8 (3)
N2^i^—Nd1—N3—C17	129.96 (18)	C9—C10—C18—C17	177.2 (3)
O2—S1—C1—C2	11.2 (3)	C11—C10—C18—C17	−2.0 (4)
O3—S1—C1—C2	133.4 (2)	N3—C17—C18—N2	−0.3 (4)
O1—S1—C1—C2	−109.8 (2)	C13—C17—C18—N2	177.5 (2)
O2—S1—C1—C6	−169.6 (2)	N3—C17—C18—C10	−178.1 (2)
O3—S1—C1—C6	−47.4 (2)	C13—C17—C18—C10	−0.4 (4)

Symmetry codes: (i) −*x*+1/2, −*y*+1/2, *z*; (ii) −*x*+1/2, −*y*+3/2, *z*.

### Hydrogen-bond geometry (Å, °)

**Table:**

*D*—H···*A*	*D*—H	H···*A*	*D*···*A*	*D*—H···*A*
O9—H6W···S1	0.85	2.90	3.646 (2)	149
O9—H6W···O3	0.85	1.97	2.810 (3)	171
O8—H5W···N4	0.86	2.61	3.391 (3)	152
O8—H5W···O7	0.86	2.53	3.109 (4)	126
O8—H5W···O6	0.86	1.99	2.842 (3)	170
O8—H4W···S1	0.83	2.93	3.661 (3)	148
O8—H4W···O3	0.83	2.03	2.861 (3)	174
O9—H7W···N1^iii^	0.86	2.24	2.982 (4)	145
O5—H3W···O8^i^	0.85	1.96	2.793 (3)	168
O5—H2W···O9^iv^	0.85	1.83	2.670 (3)	171
O4—H1W···O2^iv^	0.83	1.95	2.773 (3)	171
N1—H1A···O8^v^	0.89	2.29	3.127 (4)	158

Symmetry codes: (iii) *x*−1/2, −*y*+1, −*z*+1/2; (i) −*x*+1/2, −*y*+1/2, *z*; (iv) *x*, −*y*+1/2, *z*−1/2; (v) −*x*+1, −*y*+1, −*z*.
